# High intensity interval training for Parkinson's disease: A scoping review of systemic effects and physiological adaptations

**DOI:** 10.1177/1877718X261427268

**Published:** 2026-03-05

**Authors:** Anjali Sivaramakrishnan, Meenal Cascella, Samantha Hauck, Noe Simon Reyes, Okeanis Vaou

**Affiliations:** 1 Motor Neuromuscular Exercise Rehabilitation Laboratory, Department of Physical Therapy, School of Health Professions, The University of Texas at San Antonio, Health Science Center, San Antonio, TX, USA; 2 Long School of Medicine, The University of Texas at San Antonio, Health Science Center, San Antonio, TX, USA; 3 Division of Movement Disorders, Department of Neurology, The University of Texas at San Antonio, Health Science Center, San Antonio, TX, USA

**Keywords:** high intensity interval training, aerobic exercise, cardiorespiratory fitness, Parkinson's disease, chronotropic incompetence

## Abstract

**Background:**

High intensity interval training (HIIT) involves vigorous intensity exercise bouts interspersed with low intensity bouts. Despite growing interest, the optimal dosage and clinical adaptability of HIIT in Parkinson's disease (PD) remain unclear. This scoping review synthesized the literature on systemic adaptations underlying HIIT in PD and developed a clinical framework while considering chronotropic incompetence, orthostatic hypotension, and disease progression.

**Methods:**

Three databases were searched for studies that incorporated HIIT interventions in PD. The Template for Intervention Description and Replication checklist was used to characterize the quality of intervention reporting.

**Results:**

A total of 285 studies were screened, of which 10 studies were included. HIIT was administered 2–3 times/week for 30–60 min/session over 8–12 weeks. Seven studies used moderate-volume HIIT and three studies used high-volume HIIT protocols. The quality of intervention reporting was fair to good. HIIT improved cardiorespiratory fitness, motor severity, and functional mobility in PD, however, improvements were comparable to moderate intensity continuous training (MICT). HIIT may facilitate neuroplasticity by increasing brain-derived neurotrophic factor levels and dopamine transporter uptake. We recommend that HIIT programs for individuals with autonomic dysfunction use individualized heart rate targets, and perceived exertion for determining exercise intensity, and incorporate longer duration programs (>12 weeks).

**Conclusion:**

HIIT is a well-tolerated intervention that may improve cardiorespiratory fitness, disease severity, and certain neurobiological markers in mild-moderate PD, with benefits similar to MICT. Larger trials comparing different HIIT volumes are needed to identify optimal exercise volume to inform individualized exercise prescription.

## Plain language summary

*High intensity interval training and Parkinson's disease*

This review studied the effects of high intensity interval training (HIIT) for people with Parkinson's disease (PD). HIIT is an exercise program that involves alternate short bursts of intense exercise followed by rest periods. Although HIIT is being explored for people with PD, its effects and clinical adaptability is not well understood. The researchers performed a literature review to obtain a detailed understanding of the effects of HIIT in PD. The researchers were also interested in finding out how HIIT can be adjusted for certain characteristics unique to PD such as autonomic nervous system problems and progressing disease.

The reviewers found 285 studies when they ran the search and found 10 that met eligibility criteria. HIIT programs in these studies were performed twice or thrice per week, for 30–60 minutes over 8–12 weeks. Their findings showed that HIIT can improve fitness (i.e. ability to exercise for longer durations without getting tired), movement difficulties such as slowness, shakiness or stiffness and the ability to move around. It may improve brain health by increasing levels of a protein related to learning and growth, and a chemical that manages dopamine levels. However, HIIT did not seem to be more beneficial compared to moderate-intensity exercise. Overall, HIIT appears safe and effective for increasing fitness and movement difficulty for people in the early to middle stages of PD. More research via larger trials is needed to compare different types of HIIT protocols to understand how to integrate it into treatment protocols for PD.

## Introduction

Parkinson's disease (PD) is a progressive neurodegenerative condition which is projected to affect 25 million people globally by 2050.^1^ Aerobic exercise, broadly defined as continuous rhythmic activity that increases heart rate and breathing to sustain prolonged effort,^2^ has emerged as an intervention that improves non-motor symptoms such as cognition and may slow disease progression.^3–6^ Traditional aerobic exercise includes moderate intensity exercise [64–76% maximal heart rate (HR_max_)] and high or vigorous intensity exercise (≥77% HR_max_).^7^ Recent evidence from preclinical models and human studies suggests that high intensity aerobic exercise is potentially neuroprotective and shows promise for slowing disease progression.^8–11^

While opting for high intensity exercise is preferred due to its disease modifying potential, implementing such protocols into clinical practice can be challenging. Ongoing clinical trials are comparing whether high intensity continuous training (HICT) is superior to moderate intensity continuous training (MICT).^12^ However, HICT programs require individuals to sustain vigorous intensities for long durations over several months, which can become monotonous and affect long term adherence. While MICT is more tolerable than HICT, it provides a weaker physiological stimulus compared to high intensity protocols. Notably, individuals with PD who have more impairments may tolerate higher exercise intensities only through interval-based protocols.

High intensity interval training (HIIT) is a time-efficient protocol that involves bursts of vigorous intensity exercise which are at near maximal oxygen uptake (VO_2max_) i.e., (approximately 80–90% VO_2max_ or 85–95% HR_max_),^13^ interspersed with low intensity recovery bouts or rest periods. HIIT can induce physiological adaptations in the cardiopulmonary and vascular systems and promote skeletal muscle metabolism.^14^ HIIT can elicit both central and peripheral adaptations, which can increase VO_2max_.^15^ Although these effects overlap with continuous protocols, meta-analyses in individuals with obesity and metabolic syndrome show that HIIT elicits greater improvement in cardiorespiratory fitness (CRF),^16^ body composition and metabolic health,^17^ while requiring lower total exercise volume and energy expenditure compared to MICT.^18^ Additionally, HIIT may improve adherence as it is perceived to be more enjoyable than MICT.^19^ HIIT protocols are therefore advantageous as the exercise durations are shorter, making it more suitable for individuals with PD who present with greater motor severity or other comorbidities which may preclude them from performing high intensity continuous exercise.

There is growing interest in implementing HIIT in PD. A recent systematic review by Harpham et al. included eleven articles and reported that HIIT is safe and can elicit clinical improvements in CRF, brain-derived neurotrophic growth factor (BDNF) levels and motor symptoms for individuals with mild to moderate PD.^20^ HIIT could be an ideal candidate for neuroprotection as animal studies also show that HIIT can increase BDNF and facilitate TrkB receptor expression,^11,21^ potentially improving dopaminergic neuronal survival and motor function. While these early findings are promising, there remains substantial heterogeneity in HIIT dosage. This scoping review builds upon the recently published systematic review,^20^ by classifying HIIT protocols according to their dosage and discussing the systemic and clinical effects.

HIIT is commonly prescribed as a one-size fits all approach, however it is important to consider other PD-specific characteristics that may influence its safety, effectiveness and disease modifying potential. Here, we discuss three considerations, namely, chronotropic incompetence (CI), orthostatic hypotension (OH), and disease progression. These considerations can directly affect cardiovascular and hemodynamic responses,^22,23^ exercise capacity,^24^ and the risk for developing adverse events. Autonomic dysfunction is reported even in early PD stages.^25^ The cardiac postganglionic sympathetic outflow can be affected, potentially causing lower blood pressure (BP) and heart rate in response to maximal exercise testing, also termed as chronotropic incompetence.^26^ Orthostatic hypotension is another autonomic impairment that can cause dizziness and syncope and interfere with daily functioning. Other symptoms that may interfere with exercise include impaired postural reflexes that may not be evident in the earlier stages but become more prevalent with disease progression. We selected these factors as growing evidence suggests that autonomic dysfunction and disease severity can influence exercise performance.^24,27^ Moreover, patients with PD frequently identify autonomic impairment, such as OH, as highly disabling with a negative impact on their quality of life.^28^ Taken together, HIIT protocols may need ongoing adjustments based on factors unique to PD.

Therefore, in this scoping review, we aim to: (i) provide an updated summary of the physiological and clinical effects of HIIT across multiple systems in PD while drawing upon literature from other clinical populations, (ii) explore how considerations such as autonomic impairment and disease progression may guide the prescription of HIIT protocols.

## Methods

This review was conducted according to the Preferred Reporting Items for Systematic Reviews and Meta-Analyses extension for scoping reviews (PRISMA-ScR) guidelines,^29^ and follows the methodological framework proposed by Arksey and O’Malley,^30^ and updated by the Joanna Briggs Institute.^31^ The five stages are described below.

### Identifying the research question

The scoping review addressed the following key research questions:

- What are the systemic and clinical effects of HIIT in PD?
- How can HIIT be adapted to people with PD who present with CI, OH or disease progression?

### Identifying relevant studies

A comprehensive search was performed in PubMed, Scopus and CINAHL with no restrictions on publication date (most recent search: 9 September 2025). Search strategies included index terms, medical subject headings (MeSH) terms and keywords related to “high intensity interval training”, “endurance training”, “aerobic exercise”, “walking”, “cycling”, “ergometry”, “treadmill”, “resistance training” AND “Parkinson's disease” or “Parkinson's”. See Supplementary Table S1 for search strategy.

### Study selection

We included studies that: (i) were randomized or non-randomized studies with at least one intervention arm being HIIT, (ii) incorporated single or multiple HIIT sessions, (iii) had ≥5 participants, (iv) included outcomes pertaining to cardiovascular function, disease severity, functional outcomes such as cognition, strength, endurance and molecular markers such as neurotrophins and anti-inflammatory markers. The American College of Sports Medicine (ACSM) criteria were used to define exercise intensity for the interval bouts.^7^ Vigorous intensity referred to 77–95% HR_max_ / 64–90% VO_2max_/ rate of perceived exertion (RPE) 14–17/ 60–89% HR reserve.^7^ We excluded studies: (i) in other populations, (ii) used only moderate intensity continuous or interval exercise or HICT, (iii) non-English articles, (iv) conference proceedings, (v) protocol papers, (vi) animal studies and (vii) case reports. All articles published in English with no restrictions on date were included. Title and abstract screening were independently performed by two reviewers followed by full text screening with any discrepancies that were resolved by mutual discussion.

### Data charting

Data extraction included the following: (i) type of study, (ii) characteristics of the population, (iii) HIIT dosage according to frequency, intensity, time, type, volume and progression - FIITVP, (iv) characteristics of outcomes such as VO_2max_, 6-min walk test (6MWT), Unified Parkinson's Disease Rating Scale (UPDRS)-III score, cognition, etc. and biological outcomes including BDNF, dopamine transporter protein (DaT) and other biomarkers and (v) major findings related to clinical/neurophysiological or behavioral outcomes (Table 1). HIIT protocols were classified by session volume (low: < 5 min, moderate: 5 to <15 min, high: ≥15 min at vigorous intensity), interval duration (sprint: 10–30 s at near-maximal intensity; short: <30 s; moderate: >30 s to <2 min; long: ≥2 min at sub-maximal intensity), and intervention length (short-term: <4 weeks; moderate-term: 4 to <12 weeks; long-term: ≥12 weeks).^32^ Table 2 shows a heat map representation of HIIT protocols across studies.

**Table 1.** Characteristics of included studies.

Author	Sample (n, M/F), age (Y), PD characteristics	Study design	Groups	Mode, exercise testing	Frequency, intensity (% HR_max_, RPE), time (minutes), type, volume, progression	Outcomes	Main findings
de Laat et al.	10 (6/4), 64.2 ± 5.2, H&Y: 2 MDS-UPDRS III: 27.6 ± 4.4	Pre-post	EG: HIIT	Multimodal ET: not performed	F: 12x/month×6 months I: 80% HR_max_ T: 60 min T: Circuit training - strength, cardio, power V: High P: Unknown	DAT, neuromelanin, MDS-UPDRS III	↑ DAT in SN and putamen ↑ neuromelanin in SN ⌀ MDS-UPDRS III
Fernandes et al.	21 (13/8), 68.6 ± 8.3, H&Y: 2	RCT	EG: HIIT C: MICT	Jogging/running ET: not performed	F: 3x/wk×12 wks I: EG: RPE 15-17 C: RPE 11-14 T: 30 min T: EG: Jogging/running C: Walking/jogging V: EG: Moderate volume C: Continuous P: Unknown	HR, BP, PWV, HRV, endothelial reactivity, TUG, 6MWT, STS	EG: ↑ 6MWT compared to control and compared pre/post EG and C: ↓ STS compared pre/post EG and C: ⌀ endothelial reactivity, TUG, HR, BP, PWV, HRV
Fiorelli et al.	12 (6/6), 66.5 ± 8, H&Y: 1.8, UPDRS-III: 24.5 (7.4)	Crossover RCT	EG1: HIIT EG2: MICT C: Rest	Cycle ergometer ET: not performed	F: Single session I: EG1: RPE 15–17 EG2: RPE 11- 13 T: EG1: 25 min EG2: 30 min T: Cycle ergometer V: EG1: Moderate volume EG2: Continuous P: N/A	Weschler Adult Intelligence Scale-II -associated verbal pairs (auditory memory), symbol search, digit span (attention) & trail making test A (sustained attention) & B	EG1: ↑immediate auditory memory, attention, sustained attention EG2: ↑ immediate and late auditory memory EG1 and EG2: ↑ sustained attention post MICT EG1 and C: ⌀ changes EG2 and C: ↑ immediate auditory memory and late memory post MICT
Haas et al.	6 (4/2), 63 ± 7.3, H&Y: 1–4	Pre-post	EG: HIIT	Cycling ET: maximal GXT, cycle ergometer	F: 2x/wk×3 wks I: >75% HR_max_ T: 25 min T: Cycle ergometer V: Moderate volume P: ↑ training load	UPDRS, MIP, strength (knee flexor, extensor), PDQ-39, Phone-FITT questionnaire, TUG, VO_2max_, HR_max_, cycle endurance	↑ cycle endurance ⌀ UPDRS, MIP, PDQ-39, Phone-FITT, HR_max_, VO_2max_, TUG, strength
Harpham et al.	13 (8/5), EG: 61.8 + 9.4 C: 65.2 ± 2.3 H&Y:1.4 (EG), 1.8 (C)	RCT	EG: HIIT C: Usual care	EG: Multimodal ET: maximal GXT, cycle ergometer	F: 3x/wk×12 wks I: >75% HR_max_ T: 32 min T: Multimodal V: Moderate volume P: ↑ training load	BDNF, VO_2max_, MDS-UPDRS-III, 30 s STS, OxPAQ	↑ 30 s STS ⌀ BDNF, VO_2max_, MDS-UPDRS-III, OxPAQ
Harvey et al.	20 (12/8) EG: 68 ± 7.8, C: 69 ± 6, H&Y: 2.2 ± 0.6	RCT	EG: HIIT C: Waitlist control	EG: Resistance training ET: maximal GXT, recumbent cycle	F: 3x/wk×12 wks I: >85% HR_max_ T: 45–60 min T: Whole body resistance training with machine V: High volume P: ↑ number intervals (1/4 wks)	VO_2max_, 6MWT, PDQ-39, MoCA	EG: ↑ VO_2max_ pre-post EG: ⌀ 6MWT, PDQ-39, MoCA pre/post EG and C: ⌀ VO_2max_ and other outcomes
Kathia et al.	28 (17/11), EG: 66.6 ± 9.5, C: 68.7 ± 5.5, H&Y: 1.9 ± 0.2 UPDRS-III: EG: 34 ± 9, C: 39 ± 9	RCT	EG: HIIT C: MICT	Cycling ET: maximal GXT, cycle ergometer	F: 3x/wk×10 wks I: EG: 94% ± 2% HR_max_, ∼90% peak power output C: 81% ± 6% HR_max_, ∼60% peak power output T: 40 min T: Stationary cycling V: EG: Moderate volume C: Continuous P: EG: ↑ power output (∼5% each week) C: ↑ cycling duration (up to 50 min)	VO_2max_, knee extensor fatigability, gait, balance, UPDRS-III, PFS-16, PWV	EG: ↑ knee extensor endurance pre-post C: ⌀ all outcomes pre-post EG and C: ⌀ VO_2max_ cycling, knee extensor fatigability, UDPRS-III, PFS-16, gait, balance, PWV
Martinez et al.	15 (9/6) 69.87 ± 5.63, H&Y: ≤ 2	RCT	EG1: HIIT (7, 4/3) EG2: HVCT (8, 5/3)	EG1: Resistance training EG2: Resistance training ET: not performed	F: 2x/wk×8 wks I: EG1: RPE 8–9/10 EG2: Power (unspecified) T: 45 min T: EG1: Bodyweight and free-weight exercises EG2: Resistance machine exercises V: EG1: Moderate volume EG2: High velocity P: EG1: Unspecified EG2: ↑ load	6MWT, 10 mW, HRR, NMSS, PDQ-39, HRV, power, swing time, estimated VO_2max_ via 6MWT	EG1 and EG2: ↑ power, ↑ swing time, ↓ 10 mW, pre-post EG1 and EG2: ⌀ 6MWT, HRR, NMSS, PDQ-39, HRV, estimated VO_2max_ when compared pre-post or between groups
O’Callaghan et al.	44 (26/18) EG1: 68.8 ± 7.9, C1: 69.0 ± 6.6, EG2: 70.4 ± 7.2, C2: 64.6 ± 8.6, H&Y: I-3	2 individual RCTs	EG1: HIIT (9, 5/4) C1: control (8, 4/4) EG2: MICT (13, 9/4) C2: control (14, 8/6)	EG1 and EG2: Resistance training C: No intervention ET: maximal GXT, cycle ergometer	F: 3x/wk×12 wks I: EG1: >85% HR_max_ EG2: 60–80% HR_max_ T: 45–60 min T: EG1: Whole body resistance training EG2: Aerobic and resistance exercises V: EG1: High volume EG2: Continuous P: EG1: ↑ intensity, ↑ repetitions (4 to 6) EG2: ↑ intensity, ↑ duration of aerobic exercises (4 to 5 min)	BDNF	EG1: ↑ BDNF EG2: ⌀ BDNF C1: ⌀ BDNF C2: ⌀ BDNF
Uygur et al.	14 (10/4) 62.6 ± 8.81, H&Y: 2.5 ± 0.6 UPDRS-III: 17.5 ± 6.4	Pre-post	EG: HSIT	Cycling ET: not performed	F: 2x/wk×12 wks I: 80% HRmax, RPE∼14 T: 30 min T: Stationary cycling V: High speed-low resistance interval P: ↑ RPM	UPDRS-III, UPDRS_Brady, H&Y, ABC, SF36, 10 mW, Steps, TUG, FRT, 4SST, 9HPT, SRT, CRT, Grip	↓ UPDRS-III, ↓UPDRS_Brady, ↓ 10 mW, ↓Steps, ↓ TUG, ↑ FRT, ↓ 4SST, ↓ 9HPT, ↓ SRT ⌀ H&Y, ABC, SF36, CRT

ABC: activities-specific balance confidence; AE: aerobic exercise; AR: accuracy rate; avg: average; BDI-II: Beck's Depression Inventory Part II; BDNF: brain-derived neurotrophic factor; BP: blood pressure; C: control; CO: cardiac output; CRT: choice reaction time; DAT: dopamine transporter; DBP: diastolic blood pressure; EG: experimental group; ET: exercise test; FIIT-VP, frequency, intensity of HIIT bout/MICT, time, type, volume and progression; FRT: Functional Reach Test; GXT: graded exercise test; Grip: grip strength; HIIT: high intensity interval training; HR: heart rate; HRmax: heart rate maximum; HRR: heart rate recovery; HRV: heart rate variability; HSIT: high speed interval training; HVCT: high velocity circuit training; H&Y: Hoehn and Yahr stage; M/F: males/females; MoCA: Montreal Cognitive Assessment Scale; MICT: moderate intensity continuous training; mins: minutes; MIP: maximum inspiratory pressure; MDS-UPDRS III: Movement Disorder Society Unified Parkinson's Disease Raing Scale; NMSS: Non-Motor Symptom Scale; OxPAQ: Oxford participation and activities questionnaire; PD: Parkinson's disease; PDQ-39: Parkinson's Disease Questionnaire; PFS-16: Parkinson's Fatigue Scale; PWV: pulse wave velocity; RCT: randomized control trial; RPE: rate of perceived exertion; RPM: revolutions per minute; RT: reaction time; SBP: systolic blood pressure; SF36: Short Form-36 health survey; SN: substantia nigra; SRT: simple reaction time; Steps: steps taken in 10 mW; STS: sit-to-stand test; SV: stroke volume; TPR: total peripheral resistance; TUG: timed up and go test; UPDRS: Unified Parkinson's Disease Rating Scale; UPDRS_Brady: UPDRS bradykinesia subsection; UPDRS-III: UPDRS Part III (motor subsection); VO_2max_: maximal oxygen consumption; wk: week; wks: weeks; 4SST: Four Square Step Test; 6MWT: 6-min walk test; 9HPT: Nine Hole Peg Test; 10 mW: 10-meter walk test; ⌀: no change. Population characteristics are expressed as mean (S.D.)/range.

**Table 2.**
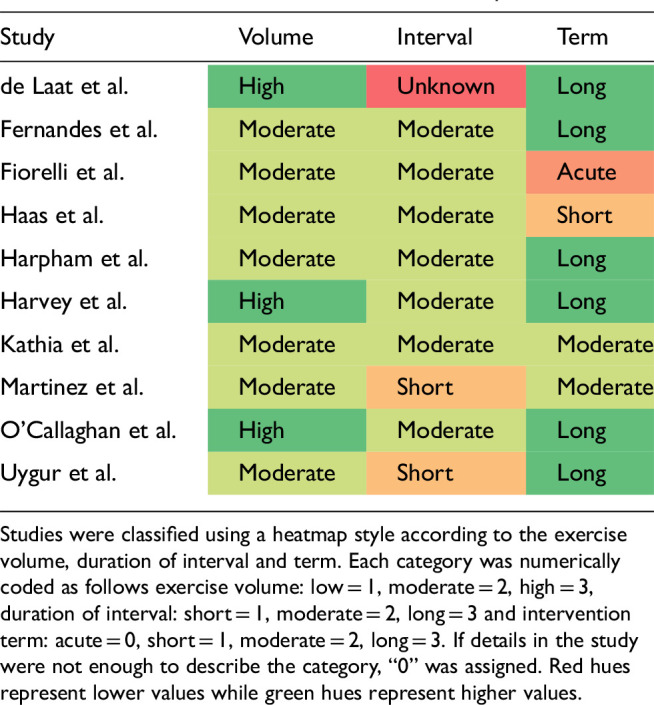
Classification of studies based on HIIT protocol.

*Data quality*: The Template for Intervention Description and Replication (TIDieR) checklist was used to characterize the quality of intervention reporting.^33^ Each question was rated with one point (maximum possible score of 12) if a clear description was provided. No points were provided if there was insufficient information.

### Data synthesis

Study design, characteristics of the population and intervention, clinical, physiological and behavioral outcomes and adverse events were summarized descriptively for the studies (Table 1).

## Results

### Search results

**Figure 1.**
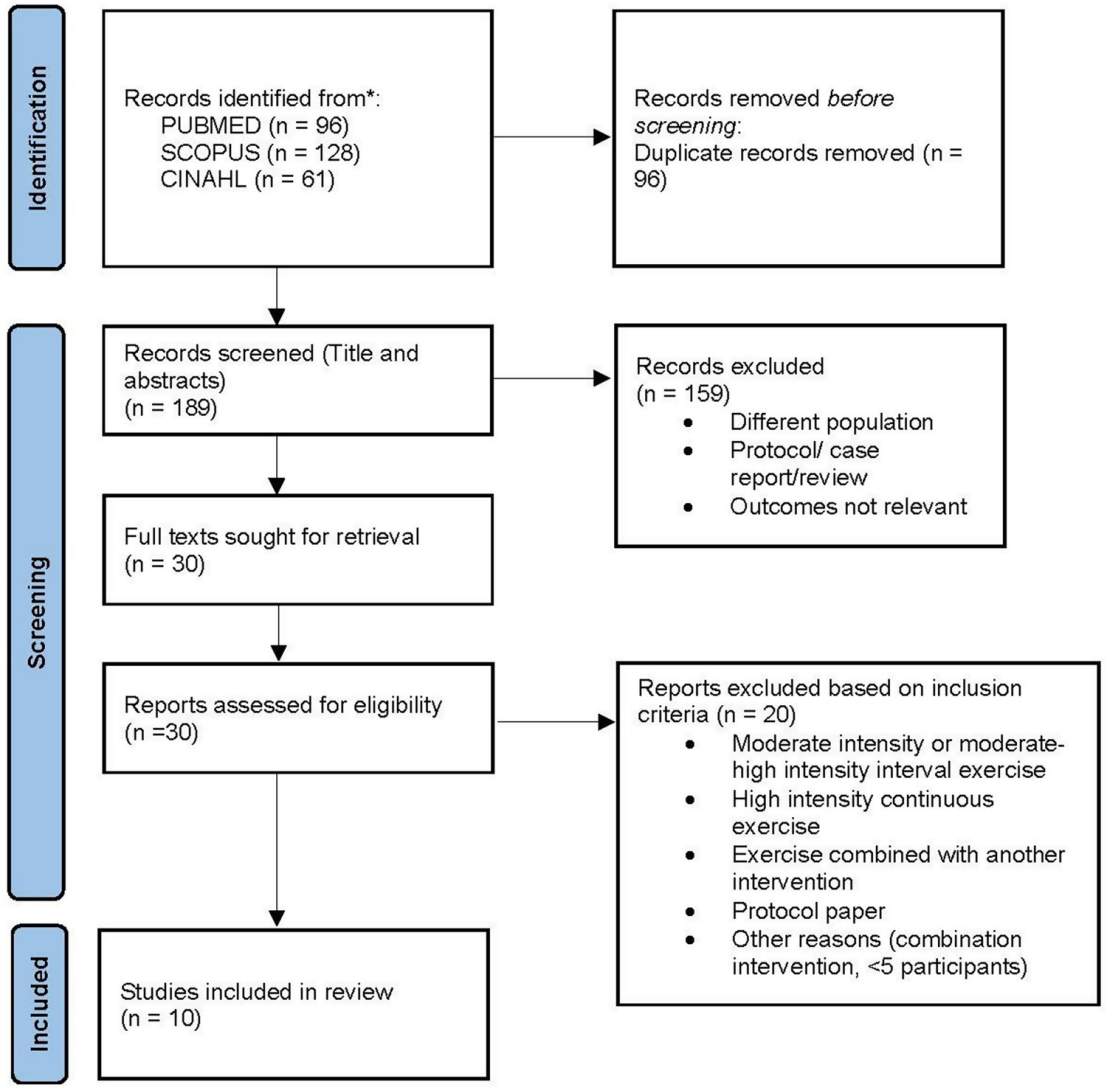
Preferred reporting items for systematic reviews and meta-analyses – scoping reviews (PRISMA-ScR) flowchart.

A total of 285 research articles were screened. After duplicates were removed (n = 96), 189 titles and abstracts were screened. 159 articles were excluded, and 30 full texts were reviewed. Twenty articles were excluded, and 10 articles were included in the review. shows the PRISMA-ScR flowchart for the study. (Figure 1)

### Characteristics of included studies

There were 183 total participants (61% males, 39% females) in the included studies. Across studies, participants were generally in Hoehn and Yahr (H&Y) stage ≤ 3, except one, which included individuals up to stage 4.^34^ Most studies had sample sizes of ≤ 20 participants,^10,34–39^ while three studies had > 20 participants.^40–42^ Six studies were randomized controlled trials (RCT) that compared HIIT to control groups that were either usual care or MICT or high velocity circuit training or no intervention.^35,36,39–42^ One study was a crossover RCT that compared single sessions of HIIT, MICT and rest conditions,^38^ and three studies consisted of single group pre-post designs.^10,34,37^ Four studies assessed outcomes in ON phase of dopaminergic medication,^36,38,41,42^ whereas one study assessed outcomes during OFF phase.^10^ The remaining studies did not specify the medication phase for outcome assessment. One study implemented HIIT in a home-based setting with remote supervision,^39^ while other studies administered HIIT in directly supervised settings.

### Characteristics of the intervention

Exercise intensity prescription: Two studies used an age predicted equation for estimating HR_max_,^10,37^ three studies used RPE to estimate intensity,^36,38,41^ and five studies used a graded exercise test (GXT).^34,35,39,40,42^

Five studies used aerobic HIIT,^34,37,38,41,42^ three studies used resistance-based HIIT,^35,36,40^ and two used multimodal HIIT.^10,39^ Four studies conducted HIIT via stationary cycling,^34,37,38,42^ three used resistance training with exercise machines or body weight or free-weights,^35,36,40^ one study used jogging/running intervals.^41^ Two studies incorporated multimodal training. One used circuits that consisted of strength, cardio and power exercises in addition to boxing,^10^ and the other used home based exercise that included both aerobic and resistance training.^39^

Table 2 shows the classification of studies in terms of session volume, length of interval and training duration. Seven studies used moderate volume HIIT protocols,^34,36–39^^,41,42^ and three incorporated high volume HIIT.^10,35,40^ Interval lengths were short in two studies,^36,37^ and moderate in seven studies.^34,35,38–42^ One study used short term HIIT,^34^ two used moderate term HIIT,^36,42^ six studies incorporated long term HIIT,^10,35,37,39–41^ and one evaluated a single session of HIIT.^38^ The work-rest ratio across studies ranged from 1:1,^34,42^ 1:2,^38,41^ 1:3,^36,37^ 3:1,^39^ and 4:3.5.^35,40^ Interval length and work rest ratio could not be determined in one study.^10^

### Data quality

The TIDieR checklist was used to evaluate the characteristics of the intervention for each study (Table 3). The average (SD) score was 9.1 ± 1.9, suggesting that at least 9/12 criteria were consistently described across studies. Three RCTs had a score of 11/12 suggesting that most items pertaining to the intervention were well described.^35,39,42^ Some of the items that were not described included materials, tailoring, modifications to the intervention and fidelity.

**Table 3.** TIDier checklist.

TIDieR checklist		de Laat et al.	Fernandes et al.	Fiorelli et al.	Haas et al.	Harpham et al.	Harvey et al.	Kathia et al.	Martinez et al.	O'Callaghan et al.	Uygur et al.
**Item 1**	Name	1	1	1	1	1	1	1	1	1	1
**Item 2**	Why (rationale)	1	1	1	1	1	1	1	1	1	1
**Item 3**	What (materials)	0	0	0	0	1	1	1	0	1	0
**Item 4**	What (procedures)	1	1	1	1	1	1	1	1	1	1
**Item 5**	Who provided (disciplinary background)	1	1	0	1	1	1	1	1	1	1
**Item 6**	How (mode of delivery)	1	1	1	1	1	1	1	1	1	1
**Item 7**	Where (location)	1	1	1	1	1	1	1	1	1	0
**Item 8a**	When and how much (frequency)	1	1	1	1	1	1	1	1	1	1
**Item 9**	Tailoring	1	0	NA	1	1	1	1	0	1	0
**Item 10**	Modifications	NA	NA	NA	NA	NA	NA	NA	NA	NA	NA
**Item 11**	How well-planned (adherence)	1	1	NA	1	1	1	1	1	NA	NA
**Item 12**	How well (fidelity)	1	1	NA	1	1	1	1	NA	NA	NA
	Total Score	10	9	6	10	11	11	11	8	9	6

TIDieR elements and rating for each study.

### Adverse events with HIIT

Overall, HIIT was generally safe and feasible with minimal adverse effects. One study reported a death in the HIIT group unrelated to the study.^41^ Other studies reported mild adverse effects such as muscle soreness, fatigue and a drop in BP.^10,35^ Two studies reported withdrawal or loss to follow up that were unrelated to the study.^36,39^

## Responses to HIIT

**Figure 2.**
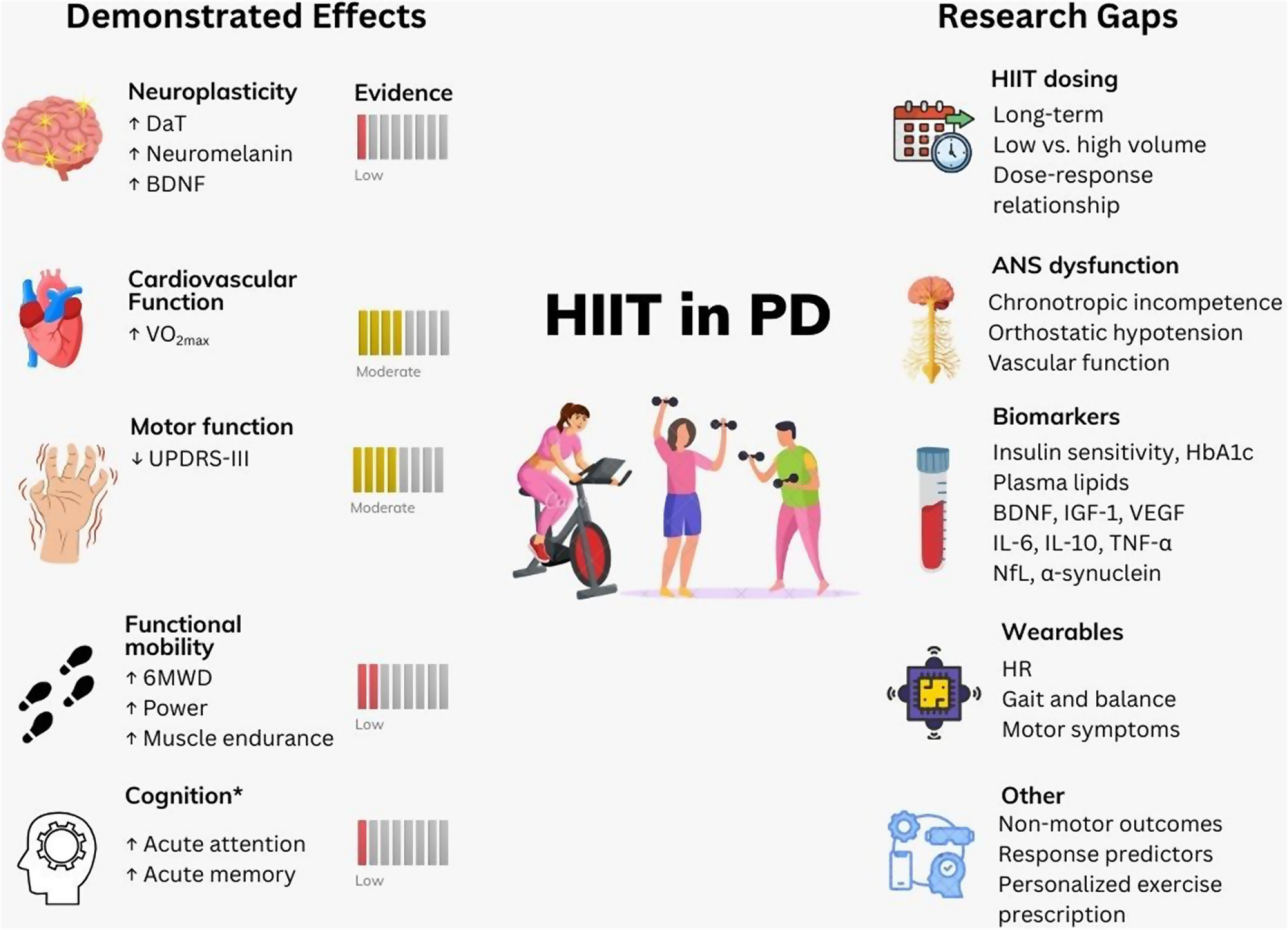
HIIT in PD: Overview of demonstrated effects of HIIT in PD (left panel) and research gaps (right panel). Evidence strength is indicated as low (red bars) or moderate (yellow bars) for demonstrated effects. The study design and number of studies per category were used to quantify the strength of effects. Abbreviations: 6MWD; 6-min walk distance, ANS; autonomic nervous system, BDNF; brain-derived neurotrophic factor, DaT; dopamine transporter, HIIT; high intensity interval training, HR; heart rate, IGF-1; insulin-like growth factor 1, IL; interleukin, NfL; neurofilament light chain, PD; Parkinson's disease, TNF-α; tumor necrosis factor-α, UPDRS-III; unified Parkinson's disease rating scale-part III, VEGF; vascular endothelial growth factor, VO_2max_; maximal oxygen uptake. *Indicates acute effects of HIIT on cognitive outcomes.

The following sections discuss the effects of HIIT on different systems and outcomes. (Figure 2)

### Cardiovascular system

Cardiovascular health is directly associated with PD, as poor cardiovascular health is associated with cognitive decline, dementia and an increased risk of mortality.^43^ Notably, autonomic dysfunction such as reduced heart rate variability (HRV), is associated with abnormal synchronization of the heart-brain axis,^44^ further highlighting its importance.

#### Cardiorespiratory fitness (CRF)

CRF or aerobic capacity refers to the maximum amount of oxygen that can be used by an individual during an activity or exercise.^45^ CRF is recommended as a vital sign,^46^ and a prognostic marker as a robust body of evidence has shown that it is inversely associated with the risk of developing cardiovascular disease (CVD),^47,48^ all-cause mortality,^49^ and other adverse health outcomes.^50,51^ In individuals with PD, CRF is reduced when compared to age-matched controls.^24^ Therefore, incorporating exercise for increasing CRF is important as individuals with PD who have been shown to have a higher relative risk for developing a stroke (odds ratio 1.65),^52^ and myocardial infarction (hazard ratio 1.67).^53^

Four studies measured VO_2max_ to evaluate responses to HIIT.^34,35,39,42^ Harvey et al. found a significant increase in VO_2max_ within the resistance-based HIIT group [mean (SD) pre-post change = 3.1 ± 2.5 mL*kg^−^1*min^−1^]. This change, however, was not different when compared to the usual care control group.^35^ Haas et al. performed a single group 3-week HIIT cycling study and found no changes in VO_2max_.^34^ Harpham et al. conducted 12 weeks of home-based HIIT using calisthenic exercises and found no change in VO_2max_.^39^ Kathia et al. performed 10 weeks of HIIT-cycling and found a clinically meaningful change following HIIT (3.7 ± 3.7 mL*kg^−1^*min^−1^), which was not significantly different when compared to MICT (1.7 ± 3.2 mL*kg^−1^*min^−1^).^42^

Overall, HIIT appears to improve CRF (i.e., VO_2max_), but it is not superior to MICT or control conditions possibly due to small effect sizes. The SPARX trial reported a large improvement (3.2 mL*kg^−1^*min^−1^) in VO_2max_ for HICT compared to usual care.^4^ In contrast, all studies in this review demonstrated smaller between group changes (<3.2 mL*kg^−1^*min^−1^). These findings may be explained by differences in total exercise volume, exercise intensity and length of intervention. Except for Harvey et al., all other studies used moderate-volume HIIT protocols. A meta-analysis based on healthy and obese adults reported that moderate to high-volume HIIT (5–15 min) with longer intervention durations (>12 weeks) incorporating higher frequencies (≥2 sessions/week with ≥ 40 min/session) has larger effects on VO_2max_ compared to MICT.^32^ Individuals with PD may similarly need >12 weeks of HIIT at intensities ∼90% HR_max_ or higher to induce stronger changes in CRF compared to control interventions.

### Hemodynamic parameters

#### Arterial stiffness

Arterial stiffness is a strong predictor of CVD and all-cause mortality.^54,55^ Increased arterial stiffness can increase BP and contribute to coronary ischemic disease and left ventricular hypertrophy.^54^ Carotid-femoral pulse wave velocity (PWV) is the gold standard measure for arterial stiffness,^56^ with higher PWV suggesting greater arterial stiffness. In PD, arterial stiffness has been associated with autonomic dysfunction, i.e., OH, supine hypertension and nocturnal hypertension (for definitions, see Table 4).^57^ Aerobic exercise has shown to improve vascular function and reduce arterial stiffness,^58,59^ indicating that HIIT could achieve similar benefits with shorter exercise volumes.

**Table 4.** Blood pressure abnormalities - definitions.

Term	Definition
Orthostatic hypotension	A drop in systolic BP ≥ 20 mmHg and/ or diastolic BP ≥ 10 mmHg within 3 min of standing or head up tilt of 60°.^60^
Supine hypertension	Systolic BP ≥ 140 mmHg and/or diastolic BP ≥ 90 mmHg after at least 5 min of rest in the supine position.^61^
Nocturnal hypertension	Elevated blood pressure during sleep characterized by reduced-dipping (<10%), non-dipping, or rising nocturnal BP compared to daytime mean BP.^61^

BP: blood pressure.

Two studies evaluated the effects of HIIT on PWV, and found no differences compared to MICT.^41,42^ Although HIIT showed a non-significant trend to reduce PWV, effects were comparable between groups. Overall, HIIT produces similar but not superior effects in PWV compared to MICT in people with PD. However, HIIT is effective for improving arterial stiffness in those with risk factors for CVD such as obesity,^62^ possibly via adipose tissue remodeling and reduced inflammation.^63^ Exercise training may promote mitochondrial function and biogenesis in adipose tissue, leading to the production of smaller adipocytes, modified extracellular matrix composition, and increased angiogenesis, resulting in reduced activation of inflammatory pathways.^63^ Future studies need to identify whether HIIT can improve arterial stiffness in patients with PD who present with risks of CVD or autonomic dysfunction.

#### Endothelial vasoreactivity

Endothelial vasoreactivity, a key indicator of vascular health, assessed via flow mediated dilation is impaired in individuals with risk factors for CVD. Endothelial function is reduced even in people with mild PD undergoing treatment with levodopa.^64^ Mechanisms such as mitochondrial dysfunction and oxidative stress likely contribute to endothelial dysfunction,^65^ as electron transport chain inhibition increases formation of reactive oxygen species that damage both dopaminergic neurons,^66^ and vascular endothelial cells.^67^ Lifestyle factors and PD-treatments further exacerbate endothelial function. Smoking reduces the bioavailability of nitric oxide (NO) which is important for maintaining vascular health,^68^ and dopamine agonists may inhibit angiogenesis by suppressing vascular endothelial growth factor (VEGF) signaling reducing endothelial cell proliferation and migration.^69^

Research on HIIT for endothelial dysfunction is limited, however studies from other populations suggest that HIIT can improve flow mediated dilation compared to MICT potentially through increased shear stress on blood vessel walls via NO-mediated vessel relaxation.^18^ HIIT-induced increases in flow mediated dilation are associated with increases in BDNF and insulin-like growth factor (IGF-1) levels.^70^ Intact endothelial function and normal IGF-1 levels are both important for neurovascular coupling, the process that regulates cerebral blood flow in response to neuronal activity.^70^ Additionally, HIIT can indirectly influence the bioavailability of NO by lowering blood glucose levels, improving insulin sensitivity and decreasing oxidized low density lipoprotein levels.^71^ Through these adaptations in endothelial tissue, skeletal muscle and adipose tissue, HIIT is thought to improve vascular health and support more efficient neurovascular function.^71^

Only one study investigated the effects of a 12-week jogging HIIT paradigm on endothelial reactivity in PD and reported a non-significant increase in endothelial reactivity compared to MICT.^41^ The authors attribute the findings to the lowered total exercise volume, as previous research has shown significant effects on endothelial reactivity with high volume HIIT that was delivered over 16 weeks.^71^ Long term studies characterizing the effects of high volume HIIT are warranted to identify its effects on endothelial dysfunction.

#### Heart rate variability (HRV)

An overview of HRV measures and their interpretation is provided in Table 5. In this review, two studies evaluated the effects of HIIT on HRV and reported no changes following either 8 or twelve weeks of HIIT.^36,41^ Interestingly, Fernandes et al. saw a decreasing trend in high frequency HRV and an increasing trend in the low frequency and high frequency ratio following HIIT but not MICT,^41^ suggesting that HIIT could potentially increase sympathetic activity in PD. Research studies evaluating the effects of HIIT on HRV are limited, however emerging studies report that 12 weeks of exercise independent of mode can improve HRV measures of global autonomic modulation, sympathetic and vagal tone in middle-aged adults.^72^ Given that individuals with PD may have autonomic dysfunction, more research in this area is needed to identify the effects of HIIT on sympathovagal balance.

**Table 5.** Heart rate variability – measure and relevance to PD.

Topic	Key points
HRV definition	Fluctuations around average HR reflecting sympatho-vagal balance.^73^
How is it measured? Time-domain measures^74^ Frequency-domain measures^74^	Determined as the time between two consecutive R-waves (R-R interval) on an ECG using time or frequency domains.^73^
	Beat to beat index: captures fast changes in HR, short term variability Long-term variability index: captures slower changes over time
	High frequency (HF): parasympathetic (vagal) activity Low frequency (LF): combined sympathetic and parasympathetic activity LF/HF ratio: reflects sympathovagal balance
Health relevance	Higher HRV is associated with lower risk of CVD and mortality.^75^
HRV in PD	Typically reduced in both time and frequency domains.^76,77^ Increasing motor severity associated with reduction in HRV, suggesting increasing sympathetic dominance.^77^

HRV: heart rate variability; HR: heart rate; R–R interval: time between two consecutive R-waves; ECG: electrocardiogram; HF: high-frequency power; LF: low-frequency power; CVD: cardiovascular disease

### Nervous system

#### Dopamine transporter (DaT) and neuromelanin

Presynaptic DaT expression in the striatum can be evaluated with single-photon emission computed tomography (SPECT) using radioligands such as ^123^I-ioflupane.^78^ Healthy individuals usually have normal DaT levels in the caudate and putamen however those with PD or other forms of Parkinsonism show reduced DaT levels.^78^ DaT SPECT has emerged as an important biomarker, where lower striatal specific binding ratio in the caudate and putamen is associated with motor symptom severity such as bradykinesia and rigidity,^79^ and non-motor symptoms including impaired cognition and anxiety.^80^

Neuromelanin is a dark brown intracellular pigment that is abundant in the neurons of the substantia nigra. In PD, the degeneration of these neurons results in a lightened area in post-mortem sections of the midbrain. Since neuromelanin is exclusively present in human brains, its relationship with physical activity is poorly understood, as rodent models do not express neuromelanin.^81^

Recent evidence suggests that higher striatal specific binding ratio on the DaT SPECT was associated with better cognition, where physical activity partially mediated this relationship.^82^ Aerobic exercise has shown to increase dopamine release in the caudate nucleus, along with increased corticostriatal activity measured via brain imaging.^83^ Animal studies suggest that physical activity could reduce neurotoxicity in the substantia nigra and increase the expression of neurotrophic factors facilitating neuroplasticity.^84^ While physical activity is considered neuroprotective in PD, the underlying mechanisms remain unclear.

Only one study evaluated the effects of HIIT on SPECT-DaT in PD. de Laat et al. found a significant increase in DaT availability in the substantia nigra and putamen, and a higher concentration of neuromelanin in the putamen following 6 months of HIIT in 10 individuals with early PD.^10^ This study provides preliminary evidence that HIIT could enhance dopaminergic neuronal function and potentially attenuate neurodegenerative changes in PD, suggesting that DaT availability and neuromelanin could be candidate biomarkers for exercise-induced neuroplasticity, pending replication in future larger controlled studies.

#### Neurotrophic factors

BDNF is an endogenous neurotrophic protein that protects dopaminergic neurons and facilitates neurotransmission. In early stages of PD, levels of circulating BDNF are reported to be ∼59% lower when compared to controls.^85^ Decreased BDNF is associated with increased motor severity and impaired cognition.^85,86^ Both acute and chronic exercise can increase circulating BDNF levels in healthy adults,^87,88^ and those with PD.^89,90^ Studies from healthy adults suggest that vigorous intensity exercise can produce greater elevation in BDNF levels,^91^ suggesting a possible dose-response relationship between exercise intensity and BDNF. Animal models consistently report neuroprotective effects of exercise that are mediated by BDNF and other neurotrophic factors including glial derived neurotrophic factor.^92^

In this review, two studies assessed the effects of vigorous intensity exercise on BDNF. O’Callaghan et al. reported a significant within-group increase in BDNF levels following HIIT but not MICT.^40^ It is unclear whether changes following HIIT were significantly higher compared to MICT. Harpham et al. reported no differences in BDNF when HIIT was compared with usual care potentially due to a lower mean exercise intensity (∼77% HR_max_).^39^ Future studies should adequately identify dose-response relationships of HIIT on BDNF and other neurotrophins such as VEGF, IGF-1 and their associations with PD-specific clinical measures.

### Clinical measures

#### Disease severity

Five studies evaluated the effects of HIIT on disease severity as measured by the UPDRS-III or Movement Disorder Society-(MDS) UPDRS III.^10,34,37,39,42^ Uygur et al. performed a single group study where participants performed 24 sessions of HIIT with low resistance cycling over 12 weeks.^37^ They reported a significant reduction in UPDRS-III scores (∼ 3.5 points). Haas et al. report no change in UPDRS-III scores following HIIT in their pre-post study.^34^ Kathia et al. report an overall reduction following both HIIT (∼ 8.7 points) and MICT (∼ 9.5 points) with no significant differences between groups.^42^ Harpham et al. observed no change in the HIIT group compared to control.^39^ Similarly, de Laat et al. observed no change following HIIT.^10^ A decrease of 3.25 points on the UPDRS-III is suggested as the minimal clinically important difference (MCID) suggestive of an improvement in disease severity.^93^ Two studies were able to elicit a change that exceeded the MCID, suggesting that 6–10 weeks of HIIT can favorably alter disease severity, however, this change was not superior to MICT. More studies in larger samples are required to replicate these findings.

#### Mobility, gait and balance

Fernandes et al. reported a significant change in 6MWT between HIIT and MICT groups.^41^ Harvey et al. and Martinez et al. reported no change in the 6MWT following HIIT.^35,36^ A decrease in the sit to stand time was observed by Fernandes et al. following both HIIT and MICT.^41^ Harpham et al. observed an increase in the 30 s sit to stand test following HIIT, however, it is not clear if this was significant.^39^ Haas et al. reported no change in the timed up and go test.^34^ Kathia et al. reported no change in gait and balance when HIIT was compared to MICT.^42^ These findings are mixed and may be attributed to the variety of HIIT protocols that were used. Arguably, a change in gait may be elicited with interventions that incorporate walking or jogging. This may explain the significant findings in 6MWT by Fernandes et al. but not the others who incorporated interventions that were not specific to walking (e.g., resistance training, cycling). Future studies are needed to determine exercise modality-related changes on specific outcome measures.

#### Cognition

Harvey et al. reported no change in global cognition measured with the Montreal Cognitive Assessment scale following HIIT.^35^ Fiorelli et al. performed a crossover study where HIIT alternated with MICT or rest and different domains of cognition were tested.^38^ Immediate auditory memory, attention and sustained attention improved within the HIIT group, however, improvements in sustained attention were significantly higher following MICT than HIIT. Similar to other outcomes, changes in cognition following HIIT may not be superior to MICT. More studies are needed to characterize acute and chronic effects of HIIT on different cognitive domains.

#### Other outcomes

The studies included in this review used a variety of outcomes to assess the effectiveness of HIIT. No change in depression, fatigue, peak power, quality of life, strength, knee extensor fatigability, non-motor symptom scale was reported between HIIT and other control groups (Table 1).

## PD-specific factors and HIIT

In the following sections, three key considerations, CI, OH and disease progression are discussed in the context of prescribing HIIT. These considerations are in line with general principles of safe exercise prescription and are similarly applicable when prescribing moderate or high-intensity continuous exercise for people with PD.

### Chronotropic incompetence (CI)

#### Definition and causes

Chronotropic incompetence (CI) refers to an inadequate heart rate response in proportion to exercise demands during a maximal stress test.^94^ It is associated with developing CVD and increased risk of mortality.^94^ Chronotropic incompetence has been reported in both cycle ergometer and treadmill-based studies, where individuals with PD show lowered HR_max_ in response to exercise.^24,95^ While its prevalence can vary with disease severity,^96^ approximately 50% of people with PD may have CI.^97^ Other studies also report that those with PD have lower HR_max_ when compared to age matched controls.^98,99^ It is thought that CI may be caused due to autonomic dysfunction which may occur prior to onset of clinical features.^94,100,101^ An imbalance in the sympathetic and parasympathetic systems can lower peak heart rate and systolic BP during exercise,^26^ and slow heart rate recovery following exercise termination.^24^ In PD, CI is associated with a higher levodopa dose and greater motor severity.^96^

#### Determining CI

Although there are no standard criteria, CI is diagnosed when an individual cannot reach 85% of the age predicted HR_max_ on a GXT or fails to reach ≥ 80% of the heart rate reserve.^94^ The respiratory exchange ratio (RER) and HR_max_ are two parameters that need to be monitored to ensure that the individual has truly attained maximal effort during GXT. A RER >1.05 or a maximal HR >85% age predicted HR_max_ are suggested as appropriate indicators of maximal effort.^94^ In this review, only two studies,^20,42^ out of the five studies that performed GXT reported these criteria. An RER value below 1.05 or maximal HR <85% HR_max_ can suggest that either the individual may have performed submaximal exercise, or the test was terminated early. This is important to consider before determining whether the individual may have CI. Other approaches to determine CI include the expired gas analysis technique which evaluates the relationship between HR and VO_2_. This is termed as the metabolic-chronotropic relationship or the chronotropic index which is the ratio of heart rate reserve to metabolic reserve during submaximal exercise.^102^ This ratio is typically 1, and any value ≤ 0.8 suggests CI.^102^ Reporting heart rate recovery (HRR) is important, as the magnitude of the decline in heart rate following termination is known to be directly related to vagal tone.^94^ Existing cutoff values for abnormal HRR in the 1^st^, 2^nd^ and 5^th^ minute post-GXT termination are as follows: HRR_1min_ <12–21 bpm, HRR_2min_ <42 bpm, and HRR_5min_ <50 bpm.^94,103,104^ A drop in heart rate below the recommended cutoffs would suggest greater risk of CVD.^105^

#### Implications for HIIT

It is important that exercise studies set individualized heart rate targets based on the GXT prior to any training regimen including continuous exercise protocols. A medical screening prior to GXT is recommended only if indicated by the ACSM pre-participation screening algorithm.^7^ While individuals with PD-CI have shown to demonstrate ∼22% lower HR_max_ during GXT compared to those without CI and controls,^106^ this does not preclude them from exercising at high intensities.^106,107^ Findings from other populations suggest that HIIT can improve CI. A study that evaluated the effects of 16-weeks of low volume and high volume HIIT on patients post myocardial infarction taking beta blockers reported that both protocols increased HR_max_ and caused faster HRR.^103^ Moreover, the proportion of individuals with CI reduced in both low-volume and high-volume groups,^103^ which suggests that CI could respond to exercise regardless of concomitant beta-blocker therapy. Their findings are in line with another study which also reports that HIIT can drive cardiovascular adaptations by stimulating cardiac vagal activity, enabling faster HRR.^108^ Therefore, future studies in PD could include low volume or high volume HIIT for at least 16 weeks for identifying effects in sympatho-vagal balance.

Since heart rate may not increase in proportion with exercise intensity due to CI, the RPE can be used to provide an estimate of an individual's exertion level. It is imperative that the participant and observer understand the RPE scale accurately. A useful strategy would entail recording both participant and observer RPE for identifying target and recovery zones for HIIT.^109^ We suggest using the Borg 6–20 scale, where intervals would correspond to an RPE of 17–18, i.e., individuals should be breathing heavily but able to talk in short sentences.^109^ In rare instances, the observer RPE can be used instead of the participant RPE, if they cannot accurately report their level of exertion.^109^

Pharmacological considerations: Beta blockers are typically taken in the morning and have their maximal effect in a few hours. Both exercise testing and training need to account for concurrent medication. It is likely that if a GXT is conducted in the morning following beta blocker ingestion, there would be greater blunting of heart rate as opposed to a GXT in the afternoon or evening.^110^ As reported elsewhere, we suggest that during exercise, the systolic BP is within 220 mm Hg and/or diastolic BP is within 105 mm Hg.^7^ Moreover, treatment with antihypertensives can cause significant drops in post-exercise BP, so it is suggested that an extended cool down with close monitoring be incorporated.^7^

### Orthostatic hypotension (OH)

#### Prevalence and symptoms

Around 35% of individuals with PD can present with OH due to autonomic dysfunction.^111^ OH may be associated with symptoms such as lightheadedness, dizziness, blurry vision or syncope.^112^ The presence of OH in people with PD suggests autonomic dysfunction which can affect cardiovascular reflexes to change in position. OH is associated with faster disease progression and falls.^113,114^ Moreover, antiparkinsonian medication such as levodopa,^115^ and dopamine agonists can decrease BP in people with and without OH, which further increases the risk of falls.^116^ Orthostatic hypotension therefore presents an important consideration that needs to be addressed carefully before exercise prescription.

#### Implications for HIIT

It is recommended that screening for OH be performed prior to initiating any form of exercise when related symptoms are present. BP and heart rate should be measured in supine, after 3–5 min of rest and after standing for 1 min and 3 min respectively.^61,117^ It is important to ensure adequate hydration before and after exercise. The training modality may need to incorporate seated devices such as a recumbent stepper or cycling or a rowing machine. Since post-exercise hypotension can be expected, an extended cool-down period is recommended with careful monitoring of BP during recovery, especially in the initial weeks of exercise. Although not demonstrated in PD, aerobic training increases plasma and blood volumes improving orthostatic tolerance.^118^ Healthy adults with orthostatic intolerance show improved responses in heart rate, stroke volume and parasympathetic tone after 3 months of jogging exercise.^119^ Previous research also suggests that an increase in aerobic capacity could indirectly improve orthostatic tolerance.^120,121^ HIIT has the potential to improve sympathovagal balance in endurance athletes, however these findings remain to be elucidated in those with PD.^122^

### Customizing HIIT for disease progression

Table 6 provides an overview of interval training protocols for people with PD using the FIIT-VP principles and recommended exercise testing depending on disease severity. In H&Y stages 1 and 2, individuals can tolerate both moderate volume-HIIT and high volume-HIIT.^10,36,42^ Exercise intensity should be determined using a GXT and progression can be guided by repeating a GXT to measure changes in VO_2max_.^123^ Ideally timing HIIT in conjunction with the ON phase of medication is recommended during all stages. If individuals are unable to keep up with the interval bouts due to exhaustion, low volume-HIIT could be incorporated with shorter bouts. Prior to implementing HIIT for individuals with balance impairment and/or lower extremity weakness, it is essential to test balance and risk of fall before choosing the exercise modality.

**Table 6.** A guideline on interval paradigms for people with PD.

H&Y stage	Frequency	Intensity	Time	Type	Volume	Progression
1–2^10,36,42^	3x/ week	80–90% HR_max_ GXT recommended	30 min (+5–10 min warm up, cool down) HV-HIIT: 4 intervals, 2 min each MV-HIIT: 8–9 intervals, 30 s each	Treadmill, cycle, elliptical, cross trainer, resistance training, multimodal, boxing	HV-HIIT /MV-HIIT	Increase length and number of intervals (increasing speed, resistance)
3^35,37,39–41^	3x/ week Non-consecutive days	77–85% HR_max_ GXT recommended	30 min (+5–10 min warm up, cool down) LV–HIIT: 3–4 intervals, <30 s each	Recumbent stepper, stationary cycle, body weight supported treadmill, resistance training, multimodal	LV-HIIT, progress to HV-HIIT as tolerated	Reduce duration of rest periods as HR acclimates to exercise
4^124^	2x/ week	60–65% HR_max_ or RPE 11–13 Submaximal exercise test on recumbent stepper or 6MWT or 2MWT with assistive aid	15–20 min (+5 min warm up, cool down each) LV-MIIT: 1–2 intervals, ≤20 s each, >2 min rest	Recumbent stepper, seated boxing, resistance bands, treadmill with harness if tolerated	LV-MIIT	Increase exercise tolerance

2MWT: 2-min walk test; 6MWT: 6-min walk test; GXT: graded exercise test; HR_max_: Heart rate maximum; H&Y stage: Hoehn and Yahr stage; HIIT: high intensity interval training; HV-HIIT: high-volume HIIT; LV-HIIT: low-volume HIIT; LV-MIIT: low-volume moderate intensity interval training; MV-HIIT: moderate-volume HIIT.

In H&Y stage 3, a GXT is recommended preferably on a cycle ergometer,^125^ however a submaximal test can also be considered. A low volume-HIIT protocol would be better suited using seated exercise modalities with progression to high volume-HIIT as tolerated.^35,37,39–41^ These individuals may need <30 s intervals with more time for active recovery. In H&Y stage 4, submaximal testing, or the 6MWT can be considered. A moderate intensity interval training protocol incorporating low intensity recovery bouts is suggested. Since the evidence in this area is sparse, these recommendations are informed by exercise guidelines for PD that suggest low to moderate intensity exercise at this stage.^124^ Low intensity training could be a starting point for exercise training as it is considered feasible for most individuals with PD.^126^ It is important to consider safety precautions such as the use of a gait belt or harness and minimizing postural transitions. Individuals can perform short intervals as tolerated with longer recovery periods. Dyskinesias, dystonia and heat intolerance may need careful monitoring. Seated modalities such as recumbent stepper or arm ergometry are recommended to accommodate for reduced mobility and exercise tolerance.

## Research gaps

Despite growing interest in implementing HIIT protocols in PD, several research gaps remain. Most studies are small, moderate volume HIIT programs where the dosage is not adequate to obtain sustainable, meaningful effects that are superior to MICT. Future research should establish safety and feasibility of HIIT for individuals in H&Y stages 4 and 5, as the lack of current evidence for these stages precludes the recommendation of robust guidelines. Adherence to HIIT protocols outside of supervised settings is not clear, however, early findings by Harpham et al. suggest that HIIT can be feasible when administered in a home-based environment.^39^ The use of wearable technology to identify motor symptom severity, balance and gait can help identify effects of HIIT on symptoms of PD outside of a supervised setting.^127^ Long term, large RCTs are needed to identify the optimal HIIT paradigm by comparing different HIIT paradigms and their dose-response relationships.

Current HIIT-based studies have only started to integrate biomarkers to understand the mechanisms underlying exercise. It is not well understood if HIIT affects markers of metabolic health such as insulin sensitivity, glucose levels, glycosylated hemoglobin, plasma lipid levels and body composition indices. These markers are particularly relevant in people with PD as they can have abnormal glucose metabolism which can affect neuronal functioning.^128,129^ Moreover, hyperglycemia and diabetes have been shown as risk factors for developing sporadic PD.^130^ Previous research in elderly adults suggests that six weeks of HIIT can improve insulin sensitivity and reduce body fat and plasma lipids.^131^ It remains to be identified if these findings are reproducible in those with PD.

Similarly, the effects of HIIT on inflammatory markers are not well recognized. Several preclinical studies show that chronic inflammation is linked to PD-onset and progression.^132^ PD presents with an imbalance in the pro-inflammatory [increased levels of tumor necrosis factor α (TNF-α), interleukin (IL)-6 and IL-1β],^133^ and anti-inflammatory [increased IL-10,^134,135^ reduced IL-4^133^ and IL-1 receptor antagonist A] cytokines which results in chronic inflammation that may trigger neuronal cell death.^136^ Recent evidence in PD suggests that eight to twelve weeks of moderate to high intensity exercise can reduce TNF-α,^137,138^ and increase IL-10 levels.^138^ These findings are encouraging and need to be validated in larger studies that compare HIIT to other exercise protocols. Moreover, it is not established if HIIT can affect biological markers of neuroplasticity such as BDNF, IGF-1, VEGF and neurodegeneration (such as neurofilament light chain, α-synuclein, phosphorylated tau, glial fibrillary acidic protein and amyloid proteins). Other emerging biomarkers including the anti-aging protein, soluble klotho (s-klotho),^139^ and the exercise induced myokine,^140^ irisin warrant further research for their possible roles in PD. Further research is needed to identify the unique effects of HIIT on hematological markers related to sympathovagal balance and endothelial function. These markers are more relevant in PD as autonomic dysfunction can increase risk for CVD. Finally, it is unknown whether changes in molecular markers are associated with clinical improvements or disease severity. Future research should prioritize longitudinal HIIT studies that analyze a variety of biomarkers in addition to clinical outcomes. Integrating multimodal approaches with a combination of imaging, biomarker and clinical data may help identify responders to HIIT and predictors such as genetic and biomarker profiles to enable personalized exercise prescription.

## Limitations

An important limitation of our scoping review is the paucity of studies that incorporated HIIT paradigms. We excluded many studies that used moderate-high intensity paradigms. Very few studies reported the actual exercise intensity for their participants which made it ambiguous to determine whether the target intensity was truly in the vigorous intensity range. Moreover, not all studies incorporated GXT, which is necessary for identifying an individual's true HR_max_. Other methodological considerations such as the lack of blinding and outcome assessments during the ON phase affected the interpretation of data. Most studies restricted inclusion to H&Y stages 1–3, which limits evidence for advanced PD stages. There was considerable heterogeneity in outcomes, study designs and HIIT protocols which made it challenging to obtain a clear understanding of the role of HIIT in PD-rehabilitation.

## Conclusion

This scoping review suggests that HIIT is safe and feasible in individuals with mild to moderate PD in supervised settings. HIIT requires less time commitment compared to continuous training and can be a valuable exercise option for those with PD. HIIT improves CRF, disease severity and functional mobility, however these are comparable, but not superior to MICT. HIIT can acutely improve attention and memory, however it may not improve other outcomes such as mood or quality of life. HIIT does not affect hemodynamic parameters such as PWV, endothelial vasoreactivity or HRV. Small pilot studies suggest that HIIT can upregulate DaT, neuromelanin and BDNF levels, suggesting potential neuroprotective effects. There was considerable heterogeneity in HIIT protocols across studies including factors which may explain why HIIT programs were not superior to MICT. The adherence to the TIDieR checklist was fair to good across studies. We recommend using an individualized HR_max_ and perceived exertion as intensity metrics and consider administering HIIT protocols over 12–16 weeks for individuals with autonomic dysfunction.

## Supplemental Material

sj-docx-1-pkn-10.1177_1877718X261427268 - Supplemental material for High intensity interval training for Parkinson's disease: A scoping review of systemic effects and physiological adaptations Supplemental material, sj-docx-1-pkn-10.1177_1877718X261427268 for High intensity interval training for Parkinson's disease: A scoping review of systemic effects and physiological adaptations by Anjali Sivaramakrishnan, Meenal Cascella, Samantha Hauck, Noe Simon Reyes and Okeanis Vaou in Journal of Parkinson's Disease
