# Supplementary material for: High intensity interval training for Parkinson's disease: A scoping review of systemic effects and physiological adaptations
Source: J Parkinsons Dis. 2026 Mar 5;16(3):416–37. doi: 10.1177/1877718X261427268 (PMC13347610; doi:10.1177/1877718X261427268)
Supplement: sj-docx-1-pkn-10.1177_1877718X261427268 - Supplemental material for High intensity interval training for Parkinson's disease: A scoping review of systemic effects and physiological adaptations [file sj-docx-1-pkn-10.1177_1877718X261427268.docx]

High Intensity Interval Training for Parkinson’s disease: A Scoping Review of Systemic Effects and Physiological Adaptations

Supplemental Material

Table S1 Search strategy

(("parkinson disease"[MeSH Terms] OR parkinson[tiab]) AND (("high intensity interval training"[MeSH Terms]) OR (high intensity interval training[tiab]) OR (HIIT[tiab]) OR ("endurance training"[MeSH Terms]) OR (endurance training[tiab]) OR (aerobic exercise[tiab]) OR (high intensity exercise[tiab]))) AND ("neuronal plasticity"[MeSH Terms] OR Neuroplasticity[tiab] OR "neural plasticity"[tiab] OR "BDNF"[tiab] OR "brain-derived neurotrophic"[tiab] OR neurotrophin[tiab] OR "fMRI "[tiab] OR functional magnetic resonance imaging[tiab] OR "cognition"[MeSH Terms] OR cognition[tiab] OR motor function[tiab] OR "movement"[MeSH Terms] OR movement[tiab] OR cortical activation[tiab] OR motor control[tiab] OR cortical response[tiab] OR cardiovascular function[tiab] OR "muscle rigidity"[MeSH Terms] OR rigidity[tiab] OR "tremor"[MeSH Terms] OR tremor[tiab] OR "neuroprotection"[MeSH Terms] OR neuroprotection[tiab]) NOT (animals[mesh:noexp]) NOT (Review[Publication Type]) NOT ((Case studies) OR (case study) OR (case report)) NOT (Systematic Review[Publication Type]) AND (english[Filter])
